# Oxaliplatin Induced Digital Ischemia and Necrosis

**DOI:** 10.1155/2015/248748

**Published:** 2015-05-04

**Authors:** Kubilay Karabacak, Murat Kadan, Erkan Kaya, Baris Durgun, Gokhan Arslan, Suat Doganci, Cengiz Bolcal, Ufuk Demirkilic

**Affiliations:** Department of Cardiovascular Surgery, Gulhane Military Academy of Medicine, Etlik, Ankara, Turkey

## Abstract

*Introduction*. Digital ischemia is a rare complication of several chemotherapeutic medications. We aimed to present a patient with digital ischemia, secondary to a new generation chemotherapeutic drug, oxaliplatin. *Case Report*. 62-year-old woman presented to our department with severe pain, paresthesia, and distal acrocyanosis on her right hand fingertips. Her complaints started five days after the third cycle of a chemotherapy protocol consisting of 5-fluorourasil (5-FU), folinic acid, and oxaliplatin due to advanced colon carcinoma. On physical examination, hemorrhagic and partly ulcerative lesions were detected at her right hand fingertips. Radial and ulnar pulses were absent at affected side. Digital subtraction angiography revealed severe vascular resistance in the affected extremity. Iloprost trometamol treatment was started with the dosage of 1 ng/kg/min. In addition, low-molecule-weight heparin was used for preventing possible microemboli. Symptomatic relief was provided after five days, and patient was discharged on 7th day of treatment. *Discussion*. The pathogenesis of oxaliplatin induced vascular toxicity remains unclear. Endothelial damage, increased adherence of platelets, deposition of immune complexes as an immunologic effect of oxaliplatin, and hypercoagulable state may be the reason for arterial thrombosis, digital microemboli, possible digital ischemia, and their several consequences.

## 1. Introduction

Digital ischemia is a rare vascular complication such as distal arterial embolism, hypercoagulopathic state, and hemorrhagia, which are well known in patients with various malignancies. However, digital ischemic events secondary to moderate vasospasm are particularly rare in cancer patients. This kind of complications is usually related to vascular disorders and may also occur as a complication of connective tissue diseases [1].

Despite their many side effects, chemotherapeutic agents have a major role in cancer treatment. Most known side effects of these agents include myelosuppression, immunosuppression, mucositis, alopecia, and neuropathy. However, digital ischemia is not a well-known complication of these agents in the literature.

Most important etiologic factors of digital ischemia in patients with malignancy are not only the neoplasia itself but also the other procoagulant situations such as hypercoagulopathic states and the chemotherapeutic protocols [1, 2].

We aimed to present a patient with digital ischemia, secondary to a new generation chemotherapeutic drug, which has modest activity against advanced colorectal cancer [3].

## 2. Case Report

**Figure 1:**
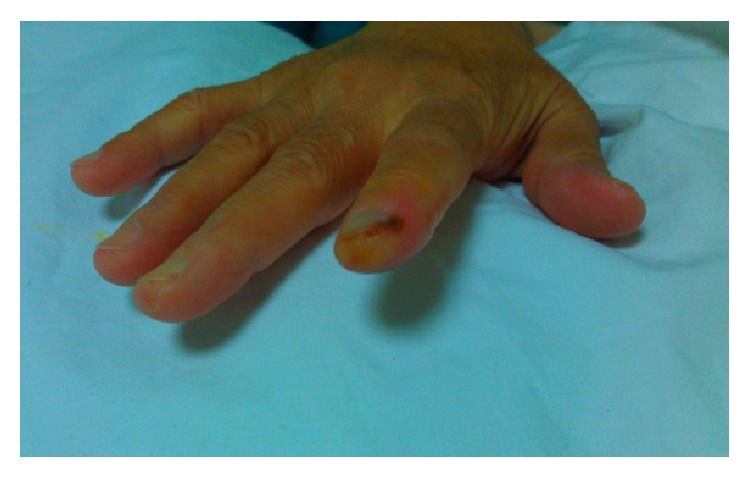
View of patient's lesions.

62-year-old female patient presented to our department with severe pain, paresthesia, and distal acrocyanosis on her right hand fingertips. Her complaints started five days after the third cycle of a chemotherapy protocol consisting of 5-fluorourasil (5-FU) and oxaliplatin due to advanced colon carcinoma. She had another chemotherapy protocol history with 5-FU alone before this protocol without any significant side effects. On physical examination, hemorrhagic and partly ulcerative lesions were detected on her right hand fingertips (Figure 1). Radial and ulnar pulses were absent at affected side. Digital subtraction angiography revealed severe vascular resistance on the affected extremity. Iloprost trometamol treatment was started with the dosage of 1 ng/kg/min. In addition, low-molecule-weight heparin was used for preventing possible microembolism. Symptomatic relief was provided after five days, and patient was discharged on 7th day of treatment.

## 3. Discussion

Colorectal cancer is the third most common cancer and the third leading cause of cancer related death in general population in the United States. Despite the improvements on diagnostic and screening techniques as well as the treatment protocols, approximately over 100.000 patients were estimated to be diagnosed with colorectal cancer in 2014 in the United States [4].

Several treatment modalities such as surgery, radiotherapy, and chemotherapy are well known for these neoplasms. One of these treatment methods or combination of them is used for that disorder, depending on the type and stage of cancer, in routine daily-practice.

The most commonly used chemotherapeutic agents for colorectal cancer include 5-flourouracil (5-FU), capecitabine, irinotecan, and oxaliplatin. FOLFOX therapy, which includes 5-FU, leucovorin, and oxaliplatin, is one of the most popular combinations of adjuvant chemotherapy protocol used for colorectal cancer.

As in all chemotherapeutic agents, these molecules also have several side effects on cardiovascular system. This kind of side effects is often seen in patients with cardiovascular risk factors such as smoking history, dyslipidemia, hypertension, diabetes mellitus, and peripheral arterial disorders. The exact etiopathogenesis of digital ischemic events is not well known in such patients, but impaired microcirculation secondary to anticancer molecules seems to be most important etiologic factor. A careful diagnostic evaluation of literature identified three possible mechanisms underlying digital ischemia: arteritis, hyperviscosity, and hypercoagulability. Thus, potential factors include endothelial damage, increased adherence of platelets, deposition of immune complexes as immunologic effects of chemotherapeutics, and hypercoagulable state.

Gemcitabine is the most well-known chemotherapeutic agent that causes several cardiovascular side effects in literature [1]. These side effects are much more common in patients with tobacco-associated cancers, especially when used in combination with platinum salt [1, 5].

Oxaliplatin, which we used in our case, acts as a potent inhibitor of DNA replication and transcription [6]. Despite its cytotoxic potency on DNA, this molecule has favorable toxicity profile. Most common side effects of this drug include neutropenia, fatigue, skin rash, ototoxicity, hypokalemia, and chemotherapy-induced peripheral neuropathy [7]. Peripheral neuropathy is a characteristic toxicity related to oxaliplatin usage that occurs in 15%–60% of patients. Its main manifestations are finger paresthesia, numbness, and tingling of the extremities often triggered by cold [8].

Another molecule that was used in our patients was 5-FU. It is a pyrimidine analogue and shows its cytotoxic effects by inhibiting the thymidylate synthase and is not a well-known molecule of chemotherapy-induced arterial occlusions [9].

Actually, the molecules used in our patient were not frequently responsible for these cardiovascular side effects in the literature, but there were not any other possible etiologic factors that can be responsible for peripheral vasotoxicity in our case. 5-FU is one of the possible etiologic agents in our case, but she did not have such side effects in her previous chemotherapy protocol, where 5-FU was used alone.

Oxaliplatin was a definite causative agent (score of 5) according to the Naranjo nomogram [10], which evaluates drug adverse events. We cannot absolutely exclude a sympathetic neurotoxic activity due to oxaliplatin leading to peripheral vasospasm. Thus, we consider that the main cause of the digital ischemia in our patient was oxaliplatin.

Independent of the etiologic molecule, treatment of the digital ischemia secondary to malignancy and chemotherapy is unclear. Iloprost treatment is advised for use in the treatment in some studies [11]. In our department, we use intravenous iloprost treatment accompanied with heparin (unfractioned or low-molecule-weight heparin) for such digital ischemic lesions, and in this case, we used the same protocol successfully.

In conclusion, we aimed to present these unusual side effects of this chemotherapeutic. In order to prevent such complications in the patient under chemotherapy, both medical crew and patient must be alert to such ischemic symptoms. Furthermore, treatment protocols must be reevaluated in case of such symptoms and suspected ischemia.

## References

[1] Kuhar C. G., Mesti T., Zakotnik B. (2010). Digital ischemic events related to gemcitabine: report of two cases and a systematic review. *Radiology and Oncology*.

[2] Taylor L. M., Michael G. H., Edwards J. M., Porter J. M. (1988). Digital ischemia as a manifestation of malignancy. *Annals of Surgery*.

[3] Nakayama G., Tanaka C., Uehara K. (2014). The impact of dose/time modification in irinotecan- and oxaliplatin-based chemotherapies on outcomes in metastatic colorectal cancer. *Cancer Chemotherapy and Pharmacology*.

[4] Siegel R., Desantis C., Jemal A. (2014). Colorectal cancer statistics, 2014. *CA: Cancer Journal for Clinicians*.

[5] Viguier J.-B., Solanilla A., Boulon C., Constans J., Conri C. (2010). Digital ischemia in two patients treated with gemcitabine. *Journal des Maladies Vasculaires*.

[6] Graham J., Muhsin M., Kirkpatrick P. (2004). Oxaliplatin. *Nature Reviews Drug Discovery*.

[7] Chay W. Y., Chew L., Yeoh T. T., Tan M.-H. (2010). An association between transient hypokalemia and severe acute oxaliplatin-related toxicity predominantly in women. *Acta Oncologica*.

[8] Raimondo L., Cella C. A., Moretto R., Matano E., Carlomagno C. (2011). Digital ischemia in patients with solid tumors: a case report and review of the literature. *Journal of Cancer Therapy*.

[9] Doganci S., Kadan M., Kaya E., Erol G., Gunay C., Demirkilic U. (2013). Acute arterial thrombosis following chemotherapy in a patient with a gastric carcinoma. *Cardiovascular Journal of Africa*.

[10] Naranjo C. A., Busto U., Sellers E. M. (1981). A method for estimating the probability of adverse drug reactions. *Clinical Pharmacology & Therapeutics*.

[11] McGrath S. E., Webb A., Walker-Bone K. (2013). Bleomycin-induced Raynaud's phenomenon after single-dose exposure: Risk factors and treatment with intravenous iloprost infusion. *Journal of Clinical Oncology*.

